# Genome-Wide Profiling of Prognostic Alternative Splicing Signature in Colorectal Cancer

**DOI:** 10.3389/fonc.2018.00537

**Published:** 2018-11-20

**Authors:** Zhen Zong, Hui Li, Chenghao Yi, Houqun Ying, Zhengming Zhu, He Wang

**Affiliations:** ^1^Department of General Surgery, The Second Affiliated Hospital of Nanchang University, Nanchang, China; ^2^Department of Rheumatology, The first Affiliated Hospital of Nanchang University, Nanchang, China; ^3^Department of Clinical Laboratory, The Second Affiliated Hospital of Nanchang University (Jiangxi Province Key Laboratory of Laboratory Medicine), Nanchang, China; ^4^Department of Cardiovascular Medicine, The Second Affiliated Hospital of Nanchang University, Nanchang, China

**Keywords:** alternative splicing, colorectal cancer, survival, splicing factor, prognosis

## Abstract

**Background:** This study was to explore differential RNA splicing patterns and elucidate the function of the splice variants served as prognostic biomarkers in colorectal cancer (CRC).

**Methods:** Genome-wide profiling of prognostic alternative splicing (AS) events using RNA-seq data from The Cancer Genome Atlas (TCGA) program was conducted to evaluate the roles of seven AS patterns in 330 colorectal cancer cohort. The prognostic predictors models were assessed by integrated Cox proportional hazards regression. Based on the correlations between survival associated AS events and splicing factors, splicing networks were built.

**Results:** A total of 2,158 survival associated AS events in CRC were identified. Interestingly, most of these top 20 survival associated AS events were adverse prognostic factors. The prognostic models were built by each type of splicing patterns, performing well for risk stratification in CRC patients. The area under curve (AUC) of receiver operating characteristic (ROC) for the combined prognostic predictors model could reach 0.963. Splicing network also suggested distinguished correlation between the expression of splicing factors and AS events in CRC patients.

**Conclusion:** The ideal prognostic predictors model for risk stratification in CRC patients was constructed by differential splicing patterns of 13 genes. Our findings enriched knowledge about differential RNA splicing patterns and the regulation of splicing, providing generous biomarker candidates and potential targets for the treatment of CRC.

## Introduction

Alternative splicing (AS) is an important post-transcriptional regulatory mechanism that regulates the translation of mRNA isoforms and generates protein diversity (1). Over 95% of human genes undergo alternative splicing and encode splice variants in the normal physiological processes (2). Emerging data demonstrated that aberrant alternative splicing events were closely associated with cancer progression, metastasis, therapeutic resistance, and other oncogenic processes (3–5). Additionally, mutations or changes in expression of splicing factors could lead to the activation of oncogenes and cancer pathways or the loss-of-function in tumor suppressors (6).

Colorectal cancer (CRC) is the third most common malignancies and the second leading cause of mortality in the United States (7). The importance of alternative splicing in colorectal cancer progression has been emphasized in many studies (8, 9). Growing evidence suggested the dysregulation of splicing events and cancer-specific spliced variants could serve as prognostic biomarkers and therapeutic targets for colorectal cancer (10, 11). Therefore, identification of the link between splicing dysregulation and colorectal cancer is a crucial issue in ongoing cancer research. Nowadays, the scarcity of studies undertake a comprehensive examination of survival associated AS events in CRC. Hence, there is an urgent need to exploit the potential prognostic value of AS events in CRC patients using the genome-wide transcriptome approach.

We illuminated the roles of differential alternative splicing patterns in 330 colorectal cancer cohort using RNA-seq data in The Cancer Genome Atlas (TCGA) program, gaining systematic insights into the potential prognostic impact of CRC-specific AS events on the survival of patients. The purpose of this study is to explore differential RNA splicing patterns and elucidate the function of the splice variants served as prognostic biomarkers in CRC. Findings in this study would help to develop the novel and appropriate therapeutic treatments of colorectal cancer.

## Materials and methods

### AS events curation from TCGA RNA-seq data

RNA-seq data of TCGA colorectal cancer cohort were available at TCGA data portal (https://portal.gdc.cancer.gov/projects). We used SpliceSeq tool, a java application, to analyze the AS profiles, and evaluate the mRNA splicing patterns for CRC patients. The Percent Spliced In (PSI) value (12), rating from zero to one, was used in quantifying AS events and calculating for seven types of alternative splicing events: Exon Skip (ES), Mutually Exclusive Exons (ME), Retained Intron (RI), Alternate Promoter (AP), Alternate Terminator (AT), Alternate Donor site (AD), and Alternate Acceptor site (AA).

### Survival analysis

Three hundred and thirty CRC patients with fully clinical parameters and at least 30 days of overall survival (OS) were included in this study. According to the median cutoff value of each parameter, patients were divided into two groups. The Cox univariate analyses were performed to estimate the clinical significance of various alternative splicing events that might influence overall survival (OS) of these CRC patients. Moreover, The Multivariate Cox regression models for independent prognostic factors were performed to candidate AS events in seven types, separately. The candidate independent prognostic AS events from seven different types were combined to build the final prognostic predictors. Furthermore, the Kaplan-Meier curves of prognostic predictor for CRC patients were compared within 5 years of OS. The chi-square test was used to compare the difference in survival status between high- and low-risk groups. Receiver operating characteristic (ROC) analyses were then used to compare the efficiency of each prediction model by survival ROC package (version 1.0.3) in R (version 3.4.3) (13).

### Integrative bioinformatics and statistical analysis

The distinguishable visualization UpSet plot, generated by UpSetR (version 1.3.3) (14), was used to quantitatively analyze the intersections between the seven types of survival associated AS events in colorectal cancer. We input the identifier names of survival associated genes into Cytocape's Reactome FI plugin (15). Gene network analyses were performed to select the critical hub genes among the survival associated AS genes. Splicing correlation network in colorectal cancer was built to represent the link between the expression of splicing factor genes and the PSI values of survival associated AS events. The correlation plots were then visualized by Cytoscape (version 3.6.0). All statistical analyses were performed using R/Bioconductor (version 3.4.3) and reported *p*-value < 0.05 was considered statistically significant(*p*-value were two-sided).

## Results

### Integrated AS events in CRC cohort

**Figure 1:**
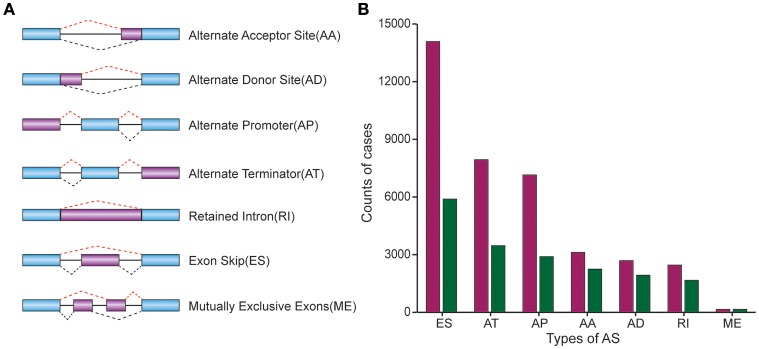
Comparison of AS patterns. **(A)** Schematic representation of AS events, Alternate Acceptor (AA) and Donor (AD) site, Alternate Promoter (AP), Alternate Terminator (AT), Exon Skip (ES), Retained Intron (RI), and Mutually Exclusive Exons (ME). **(B)** Number of AS events and related genes in CRC.

37601 AS events of 9,432 genes were identified in 330 CRC patients, suggesting the average number of AS events per gene was almost four. ES was the most frequent splice signatures among the seven AS types, followed by AT and AP. In detail, we detected 14,086 ESs in 5,890 genes, 7,939 ATs in 3,467 genes, 7,149 APs in 2,897 genes, 3,121 AAs in 2,247 genes, 2,690 ADs in 1,930 genes, 2,460 RIs in 1,666 genes, and 156MEs in 154 genes respectively (Figure 1).

### Survival associated AS events in CRC cohort

**Figure 2:**
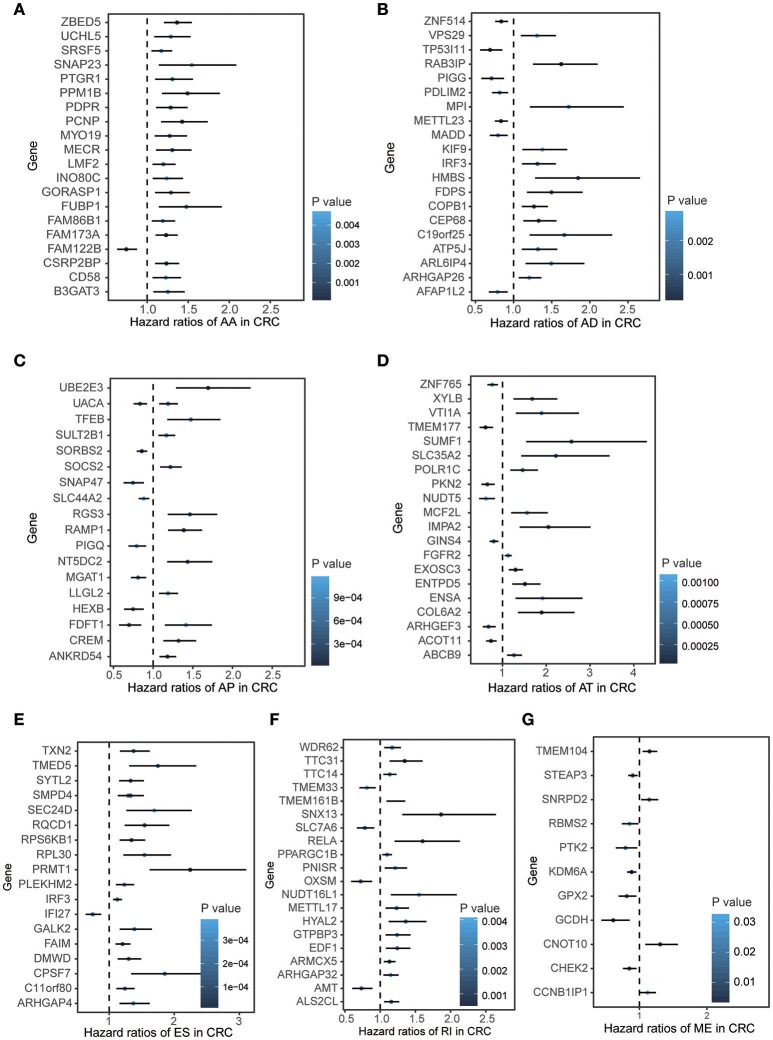
Forest plots for subgroup analyses of survival associated AS events in CRC cohort. **(A–F)** Forest plots of HRs for top 20 survival associated AA, AD, AP, AT, and RI events in CRC, respectively. **(G)** Forest plots of HRs for survival associated ME events in CRC. The color scale of the circles represents *p*-values by the side, Horizontal bars represent 95% CIs.

**Figure 3:**
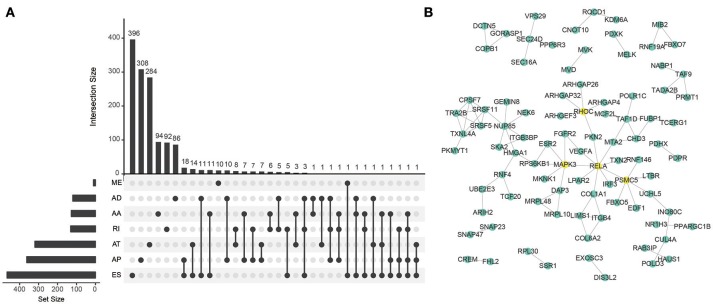
Summarize and gene network of survival associated AS events in CRC cohort. **(A)** The UpSet intersection diagram shows seven types of survival associated AS events in CRC. **(B)** Survival associated AS events interaction network created by Cytoscape.

To explore the prognostic value of AS event in CRC patients, we used the Cox univariate analyses of overall survival to evaluate the prognostic impact of each AS event. Consequently, 2,158 AS signatures were found to be significantly associated with overall survival of CRC patients (*p* < 0.05) (Supplementary Table 1). The top 20 significant survival associated AS events of the seven types were presented in Figure 2. Obviously, most of these AS events were adverse prognostic factors. Notably, one gene could have multiple survival associated AS events in CRC. Thus, a subset of overlapping AS events among the seven types of AS in CRC were illustrated by UpSet plot diagram (Figure 3A). Interestingly, most of survival associated AS genes underwent at least two types of AS, some of which even had four types of AS events. For instance, AA, AP, ES, and RI of *TCF3* were all significantly associated with overall survival. To explore the functional relationship among these significant survival associated events(*p* < 0.005), we used Reactome to generate the gene interaction networks. The results showed that the survival associated genes related to hub genes in network, such as *RELA, RHOC, PSMC5*, and *MAPK3* (Figure 3B).

### Prognostic predictors for CRC cohort

**Figure 4:**
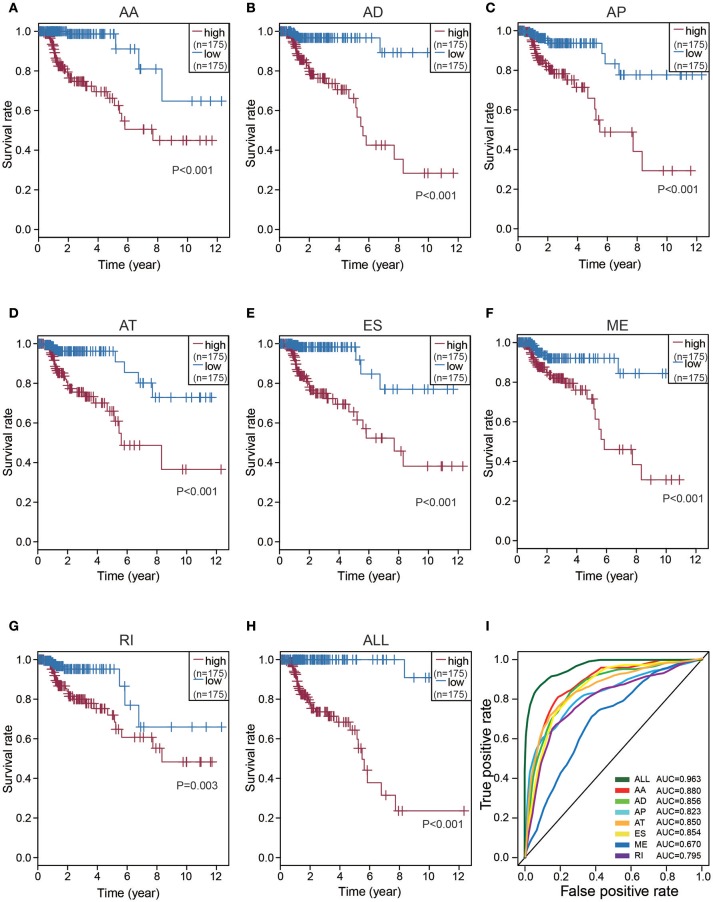
Kaplan-Meier and ROC curves of prognostic predictors in CRC cohort. **(A–G)** Kaplan-Meier plot depicting the survival probability over time for prognostic predictor of seven types of AS events with high (red) and low (blue) risk group, respectively. **(H)** Kaplan-Meier plot depicting the survival probability over time for the final prognostic predictor with high (red) and low (blue) risk group. **(I)** ROC analysis for all prognostic predictors. The color lines of ROC curves of prognostic predictors represent different types of AS events.

To detect independent prognostic factors of CRC patients, we chose the top significant survival associated AS events as candidates. The Multivariate Cox regression models for independent prognostic factors were performed to candidate AS events in seven types, separately. The candidate independent prognostic AS events from seven different types were combined to build the final prognostic predictors. In our data analysis on each type of splicing patterns, all the seven prognostic models built with different types of AS events showed significant power to predict the outcome of CRC patients (Figures 4A–G). Especially the prognostic model which was built by single AA model showed the most power to predict the outcome among the seven prognostic models (Figures 4A–G). Furthermore, the candidate independent prognostic AS events from seven different types were combined to build the final prognostic predictors. Notably, the final prognostic predictor showed better performance than each single type of splicing patterns to predict the outcome (Figure 4H). The AUC of ROC for final prognostic predictor was 0.963, followed by AA and AD model with AUC of 0.880 and 0.856, respectively (Figure 4I). Conceivably, the finally combined prognostic model had higher efficiency than other prognostic models. Additionally, 13 CRC-specific genes involved in the finally combined prognostic model were listed in Table 1.

**Table 1:** CRC-specific genes involved in the ideal prognostic model.

**Gene**	**HR**	**Lower95**	**Upper95**	***P*-value**	**Type**	**Exons**
CSRP2BP	1.343587	1.075726	1.678146	0.00923	AA	5.1
FAM122B	0.574393	0.389764	0.846481	0.005073	AA	10.1
INO80C	1.712775	1.245316	2.355706	0.000936	AA	4.1
KIF9	2.123894	1.358185	3.321288	0.00096	AD	2.2:2.3
DMWD	1.616899	1.158272	2.257123	0.004754	ES	4
PDLIM2	0.686393	0.539132	0.873879	0.002257	AD	1.2
RPS6KB1	1.390971	1.050481	1.841822	0.021236	ES	9
MECR	1.618342	1.123537	2.331059	0.009722	AA	2.1
SLC35A2	0.368709	0.152095	0.893826	0.027216	AT	6
PDPR	2.26568	1.604305	3.199706	3.42E-06	AA	13.1
ENSA	0.042622	0.009705	0.187185	2.92E-05	AT	7
EXOSC3	1.6666	1.215147	2.285779	0.00153	AT	5
FGFR2	1.437294	1.155566	1.787709	0.001118	AT	26

*CRC, colorectal cancer; HR, hazard ratio; AA, Alternate Acceptor; AD, Alternate Donor; AT, Alternate Terminator; ES, Exon Skip*.

### Network of survival-associated AS splicing factors

**Figure 5:**
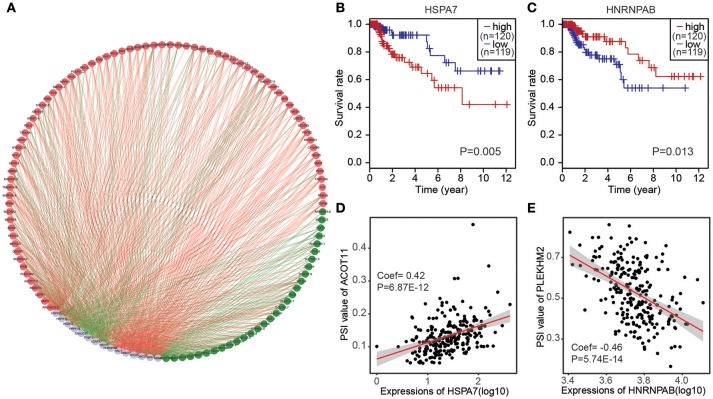
The correlation network of splicing factors and genes in CRC cohort. **(A)** Correlation network between expression of survival AS factors and PSI values of AS genes generated using Cytoscape. Gray dots were survival associated splicing factors. Green/Red dots were favorable/adverse AS events. Red/green lines represent positive/negative correlations between substances. **(B,C)** Kaplan-Meier curves for splicing factor HSPA7 and HNRNPAB with high (red) and low (blue) expression group in CRC, respectively. **(D)** Correlation between expression of splicing factor HSPA7 and PSI value of alternative splicing gene ACOT11. **(E)** Correlation between expression of splicing factor HNRNPAB and PSI value of alternative splicing gene PLEKHM2.

To determine which splicing factors were associated with the survival associated AS events in CRC, we carried out survival analysis of splicing factors based on gene expression. It was showed that 15 splicing factors were significantly associated with overall survival. Furthermore, correlations between the PSI values of top significant AS events and expression of survival associated splicing factors were investigated using Spearman's test (Figure 5A). In correlation networks, these 15 survival associated splicing factors (gray dots) were significantly correlated with 106 survival associated AS events, including 30 favorable AS events (green dots) and 76 adverse AS events (red dots). Interestingly, we found that most poor survival prognostic alternative splicing events (red dots) were positively correlated (red lines) with the expression of splicing factors (gray dots), whereas most favorable prognosis AS events (green dots) were negatively correlated (green lines) with expression of splicing factors. Splicing factors *HSPA7* was associated with worse survival of patients; Splicing factor *HNRNPAB* was linked with favorable prognosis(Figures 5B,C). Correlation between splicing factor *HSPA7* and AT of *ACOT11* were shown in dot plots, suggesting high expression of *HSPA7* were positive association with poor overall survival (Figure 5D). Similarly, correlation between splicing factor *HNRNPAB* and ES of *PLEKHM2* were shown in dot plots, implicating high expression of *HNRNPAB* were negative association with favorable prognosis (Figure 5E).

## Discussion

We identified AS events and regulatory splicing factors in colorectal cancer through the analysis of The Cancer Genome Atlas (TCGA) program to gain comprehensive insight into differential RNA splicing patterns. A total of 2,158 AS events were significantly associated with patients survival in CRC. Interestingly, most top 20 survival associated AS events of seven splicing patterns tend to carry the poor prognosis. Moreover, prognostic model was proposed by differential splicing patterns of 13 genes with high performance for risk stratification in CRC patients. Another notable finding was that distinguished splicing correlation networks between the expression of splicing factors and AS events in CRC patients.

Several studies in the exploration of AS signature have previously revealed that alternative spliced variants and cancer-specific splicing events could be served as diagnostic, predictive and prognostic biomarkers of colorectal cancer (16, 17). Recent studies reported aberrant AS events facilitated colorectal cancer development and progression through regulation of energy metabolism or DNA damage response genes (18, 19). Nevertheless, current knowledge was mostly derived from small samples studies based on exon microarray analyses by comparing colorectal carcinoma with the normal surrounding tissue (20, 21). Recently, high-throughput sequencing analysis is considered as critical technique to characterize the most common genetic aberrations of the splicesome and splice sites (22, 23). Hence, it is crucial for comprehensive understanding of the aberrant AS patterns to explore the novel therapeutic strategies for CRC.

Specifically in our data analysis on each type of splicing patterns, clinically survival benefit was observed in all the seven subgroups of splicing patterns. Observations in this study provided solid evidence that one gene could yield several mRNAs via alternative splicing(AS), resulting in multiple transcripts and various proteins, some of which even exert antagonistic functions, such as *SORBS2, VTI1A, SULT2B1*, and *ENTPD5*. This finding did not align with the previous study that reported most survival associated AS events in ovarian cancer were favorable prognostic factors (24). Conversely, colorectal cancer exhibits higher levels of adverse prognostic factors, but lower levels of favorable prognostic factors.

Upon further analysis on the prediction model built by single one type of AS pattern, Alternate Acceptor Site(AA) events showed the higher efficiency in distinguishing survival outcome of CRC patients than the predictors models built with other six types of AS pattern. Moreover, the ideal prognostic predictors model could be constructed by the combination of all seven types of AS patterns, including differential splicing patterns of 13 genes through the multivariable regression tests. The AUC of ROC for this final high-performance model could reach 0.963, performing precisely in risk stratification for CRC patients.

As one kind of molecular classification could be categorized by four distinct groups and based on their genomic signature, Consensus Molecular Subtypes (CMS) of CRC better provide prognostic information to help with the clinical management of patients and guide rational treatment with specific targeted therapies (25, 26). CRC is characterized by the set of molecular alternations, such as APC inactivation, BRAF mutation, and mutation-driven activation of K/N-RAS (Supplementary Figures 1, 2). Microsatellite instability (MSI) status along with the mutational status of K/N-RAS guide the administration of anti-EGFR (Epidermal growth factor receptor) monoclonal antibody in CRC.

Present study found the epigenetic modifications of histones near the splicing sites affected the ratio of KRAS-4A vs. KRAS-4B by the differential AS patterns in colorectal cancer cell lines (27). In a very recent study, aberrant splicing regulation of the KRAS-4A vs. KRAS-4B transcript isoforms was associated with a higher level of KRAS signaling and poor prognosis, specifically in the microsatellite stable primary CRC (28). As we have known, CRC mutant for either K/N-RAS or BRAF intrinsically resist treatment with EGFR. This study indicated the AS expands the prognostic impact of KRAS in relation to mutation status. The team of Yuan tried to modulates AS and regulate epigenetic modification of RNA processing to inhibit intestinal tumorigenesis by histone methyltransferase, which has an influence on the AS of a subset of genes implicated in tissue regeneration and tumorigenesis (29). This study aided in providing novel targeted therapeutic strategies against CRC. The next work still need further determine whether exists a link between mutational status or SNPs (Single-nucleotide polymorphism) in genes and AS events.

AS events in carcinogenesis and prognosis of CRC have been recently reported by Liu et al. (30). However, the crucial roles of splicing factors have not been well-established, emphasizing the correlation network of splicing factors and genes in CRC cohort. In addition, subgroup analyses of top 20 survival associated seven types of AS events in CRC were performed by forest plots. Moreover, we listed CRC-specific genes and relative parameters involved in the ideal prognostic model. Our findings help to investigate more prognostic AS signatures of molecular parameters in CRC cohort, which could have potential implications in highly individualized regimens to improve life expectancy based on the phenotypic characteristics and responses to treatment of CRC with different AS signatures and genes.

The interaction between alterations of splicing factors and AS events in CRC was uncovered by splicing networks analysis. With convincing data in our study that the majority of poor prognostic AS events were positively correlated with the expression of splicing factors in CRC, whereas favorable prognosis AS events were negatively correlated with the expression of splicing factors. It had been known that the process of splicing is regulated precisely by splicing factors through binding to splice-regulatory sequence elements of specific genes (31). With current knowledge that two main families of splicing factors are the Ser/Arg rich (SR) proteins and the heterogeneous nuclear ribonucleoproteins (hnRNPs) (32). SR proteins and hnRNPs always have the opposite function in the processing of RNA by AS, which bind to sequence motifs that are associated with the silencers or enhancers of splicing. This study provided a better understanding of splicing patterns and their mechanistic association to splicing factors in the CRC, which will eventually help to elucidate the underlying mechanisms of AS in oncogenesis of CRC.

Mutations or changes in expression of the regulatory splicing factors could result in aberrant splicing and differential expression of splice variants (33). Up-regulation of oncogenic splicing factors could increase the oncogenic potential of protein variants and promote colorectal cancer progression (34). More-tailored approaches for patient management, on the basis of prognostic predictors model, enabled precisely targeting of alternative-spliced isoforms and splicing regulators in CRC patients (35–37). These findings enriched our knowledge about differential RNA splicing patterns and the regulation of splicing, providing generous biomarker candidates and potential targets for treatment of CRC.

To our knowledge, this is the most comprehensive and up-to-date study to evaluate the predictors and long-term survival outcomes of CRC through molecular analysis of aberrant alternative spliced variants and regulatory splicing factors. Although the prognostic implications of these potential therapeutic targets for CRC are still need to be validated by further functional experiments and clinical trials, our study investigated the potential for the development of novel clinical biomarkers and therapeutic approaches, eventually helping to implement the novel therapeutic strategies against colorectal cancer.

## Author contributions

HW, ZZ, and ZmZ contributed conception and design of the study. HL and CY organized the database. HW Analyzed the data. ZZ and HL wrote the manuscript. HY revised manuscript. All authors contributed to manuscript revision, read, and approved the submitted version.

### Conflict of interest statement

The authors declare that the research was conducted in the absence of any commercial or financial relationships that could be construed as a potential conflict of interest.
