# Supplementary figures and images for: Genome-Wide Profiling of Prognostic Alternative Splicing Signature in Colorectal Cancer
Source: Front Oncol. 2018 Nov 20;8:537. doi: 10.3389/fonc.2018.00537 (PMC6262947; doi:10.3389/fonc.2018.00537)

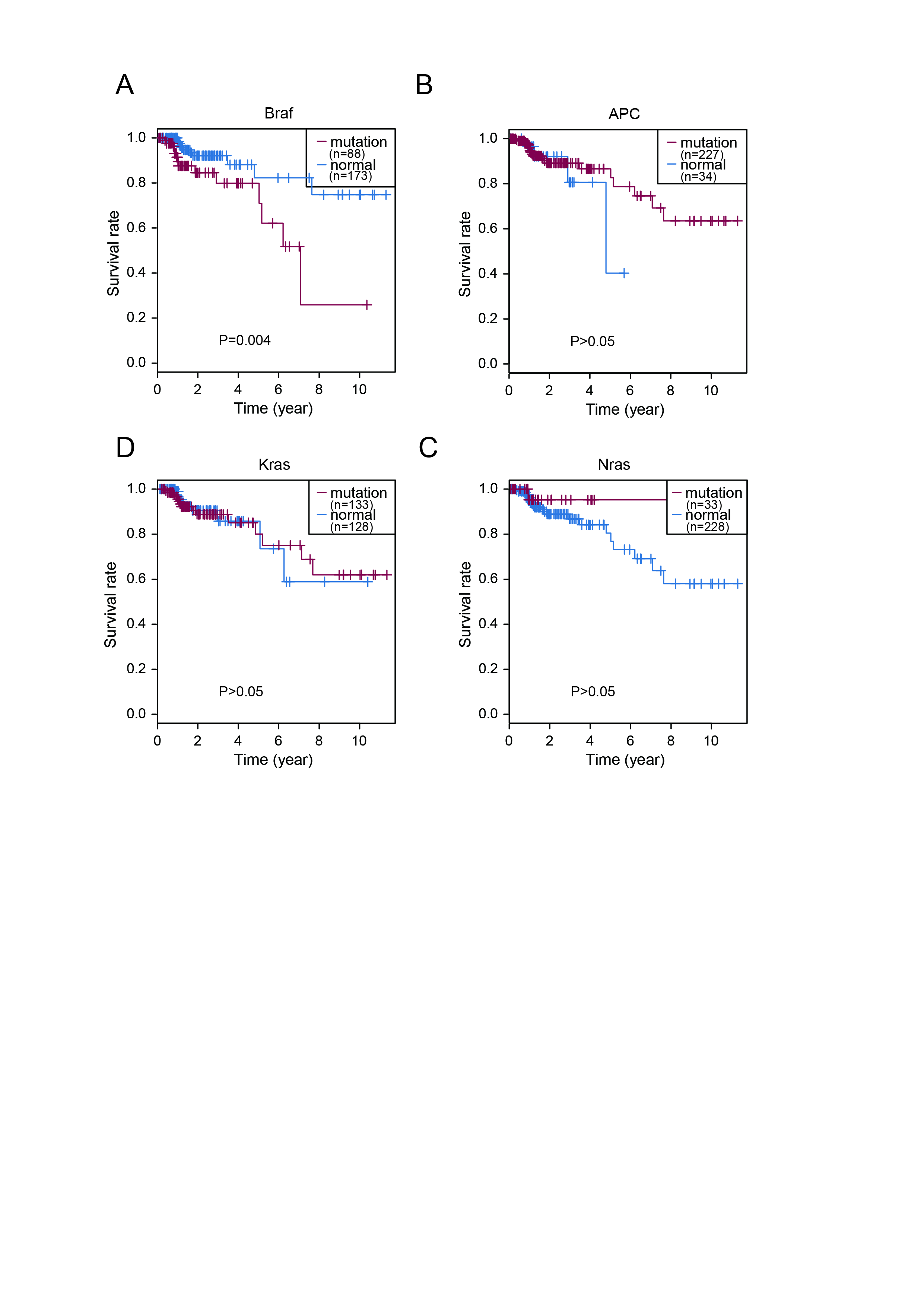

Supplement: Supplementary file 2 [file Image_1.TIF]

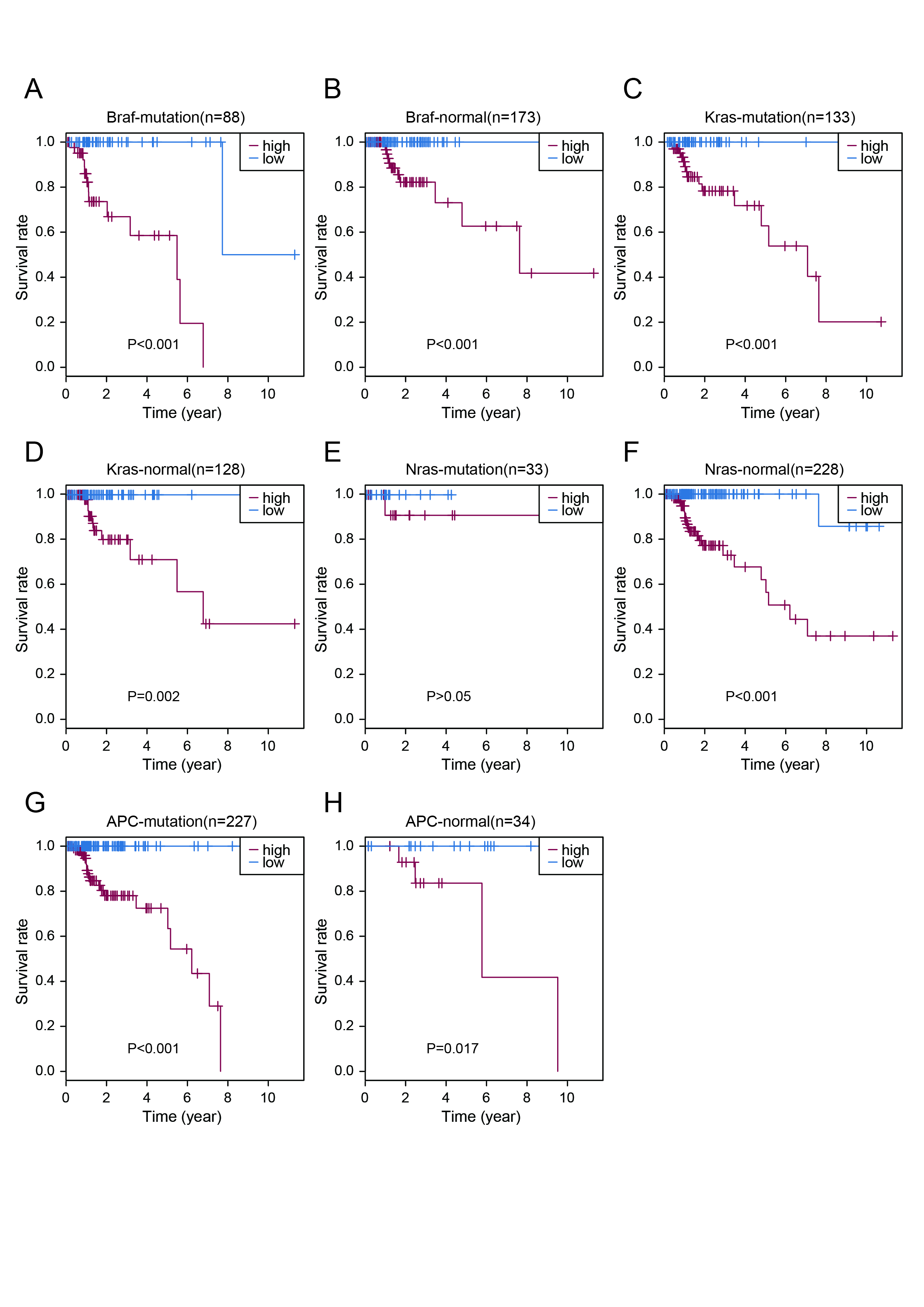

Supplement: Supplementary file 3 [file Image_2.TIF]
